# Moderate aerobic exercise training decreases middle-aged induced pathologic cardiac hypertrophy by improving Klotho expression, MAPK signaling pathway, and oxidative stress status in Wistar rats

**Published:** 2018-09

**Authors:** Behrouz Baghaiee, Pouran Karimi, Marefat Siahkouhian, Linda S Pescatello

**Affiliations:** 1 Department of Physical Education and Sport Science, Jolfa Branch, Islamic Azad University, Jolfa, Iran; 2 Neuroscience Research Center, Tabriz University of Medical Sciences, Tabriz, Iran; 3 Department of Physical Education and Sport Science, University of Mohaghegh Ardabili, Ardabil, Iran; 4 Department of Kinesiology, College of Agriculture, Health and Natural Resources, University of Connecticut, Connecticut, USA

**Keywords:** Exercise, Fibrosis, H_2_O_2_, Left ventricular hypertrophy Klotho, Mitogen-activated protein- kinase

## Abstract

**Objective(s):**

This study aimed to investigate the effect of aerobic training on serum levels of Klotho, cardiac tissue levels of H_2_O_2_ and phosphorylation of ERK1/2 and P38 as well as left ventricular internal diameter (LVID), the left ventricle wall thickness (LVWT) and fibrosis in middle-aged rats.

**Materials and Methods:**

Forty wistar rats, including young rats (n=10, 4 month-old) and middle-aged rats (n=30, 13-15 months-old) were enrolled in this experimental study. The all young and 10 middle-aged rats were sacrificed (randomly) under deep anesthesia without any exercise training as normal young control and normal middle-aged control respectively. The remaining 20 middle-aged rats participated in 4 (n=10) or 8-week (n=10) aerobic exercise training.

**Results:**

There were significant differences in the plasmatic Klotho levels and the heart tissue levels of phosphorylated-ERK1/2 (p-ERK1/2), P-P38 and H_2_O_2_, LVWT, LVID and fibrosis between young and middle-aged rats (*P*=0.01). Plasmatic Klotho level was significantly increased after eight weeks training (*P*=0.011). Also, p-ERK1/2 was significantly decreased after eight weeks and p-P38 was significantly decreased in the fourth (*P*=0.01) and eight weeks of training (*P*=0.01). A similar decrease was reported for aging-induced H_2_O_2_ in the fourth (*P*=0.016) and eighth weeks (*P*=0.001). LVID was significantly increased in eight weeks, but LVWT and fibrosis was significantly reduced in the eighth week (*P*=0.011, *P*=0.028, *P*=0.001 respectively).

**Conclusion:**

Moderate aerobic training attenuates aging-induced pathological cardiac hypertrophy at least partially by restoring the Klotho levels, attenuating oxidative stress, and reduction in the phosphorylation of ERK1/2, P38 and fibrosis.

## Introduction

Cardiac hypertrophy which refers to an increase in volume of heart occurs in some physiologic and pathologic conditions such as aging and exercise training respectively (1, 2). As the workload of the heart increases, its volume increases; a process that controls the body’s hemodynamics. This process when it occurs in healthy people particularly following participation in exercise is called physiological hypertrophy resulting in better left ventricular function (3) and differs from pathological cardiac hypertrophy. In contrary, in the case of pathological hypertrophy, the increase in the heart size eventually leads to a decrease in left ventricular function, which is an independent risk factor for heart failure (4, 5). Pathological Hypertrophy develops in two ways; Concentric hypertrophy caused by chronic pressure overload, and eccentric hypertrophy, which is caused by increased volume overload (6). There are some indicators that change their values as a result of aging and can affect pathological hypertrophy. Notably, one of these indicators is a klotho protein (7).

Anti-aging Klotho protein is produced primarily in the kidneys. Klotho is identified by a full-length type 1 transmembrane protein with an extracellular domain of 925 amino acids (the covering membrane) that has 11 intracellular amino acids in the carboxyl-terminal (8-12). *In vivo*, Klotho protein is present as a transfer membrane that is involved in the signal transmission of the fibroblast growth factor 23 (FGF23) and also identified as an endocrine (soluble in water) in blood, urine and cerebrospinal fluid (13). The soluble form of Klotho is dominant in humans and decreases with age (14). It appears to be reduced by specific signaling pathways in cardiac cells and is effective in reducing pathological hypertrophy. Cellular responses to different stimuli in cardiac cells are achieved by complex but coordinated signaling pathways. In this regard, the role of anti-aging Klotho protein in the development of Physiological Hypertrophy has received less attention.

Given that, the Mitogen-Activated protein kinase family mediates some effects of klotho protein. Recent studies suggest that klotho are able to modulate phosphorylation and thus activity of some members of mitogen-activated protein kinases (MAPKs) family such as extracellular signal-regulated Kinase 1/2 (ERK1/2) and P38 MAPK (15). ERK is a member of the MAPKs family and is identifiable as two types: ERK1 and ERK2 (16-18). ERK1/2 signaling cascade considered as a concentric hypertrophy inducing factor by activation of kinase-1 MAPKs (MEK1) in transgenic rats (19-23). 

In cultured cardiac tissue, P38 activity leads to the induction of myocyte hypertrophy and apoptosis as well as increased production of cytokines. However, a chronic activity of P38 in intact mouse cardiac tissue plays no significant role in the development of ventricular hypertrophy but instead, leads to restrictive cardiomyopathy with the remodeling of the extracellular matrix and cardiac contractile dysfunction. Furthermore, there are reports demonstrating a decline in hypertrophy in cultured tissue by increasing the activity of P38 (24-28). In general, the role of P38 in creating or reducing ventricular hypertrophy is indefinite and there is uncertainty about it. However, it can be assumed that P38 cannot be considered alone as the creator or reducer of Pathological Hypertrophy but, it is clear that changes in these pathways and markers, as result of aging, leads to extensive changes in the heart, including cardiac hypertrophy.

 Enjoying an active lifestyle can improve heart function and possibly decrease pathological hypertrophy. garciarena *et al.* (2009) reported that endurance training leads to conversion of pathological hypertrophy to physiologic hypertrophy (29). In another hand, Matsubara *et al.* (2014) reported that aerobic exercise has led to an increase of Klotho in postmenopausal women (30) and a 2007 study also showed that in response to swimming, inactivation of P38 can lead to Physiological Hypertrophy without fibrosis in rats through the elimination of ASK1 (upstream activating P38) or direct removal of the myocardium P38, but the exercise had no effect on the amount of ERK1/2 (31). Miyachi *et al* (2009), showed that P38 and ERK1/2 were significantly increased in rats with hypertension, but as a result of exercise, phosphorylation and the activity of this indicator (P38 and ERK1/2 ) declined (32). It seems that there is no comprehensive and clear view of the exact effect of aerobic exercise on the levels of Klotho protein and its relationship with ERK1/2 and P38 phosphorylation, and their association with aging-induced cardiac hypertrophy. So that, the aim of this study was to investigate the effect of eight weeks’ aerobic exercise on serum levels of Klotho, and cardiac tissue intracellular reactive oxygen species (ROS) production, triggering of MAPK pathway, fibrosis and concomitant changes in dimensions of the heart of middle-aged rats. 

## Materials and Methods

***Antibodies and reagents***

Polyclonal rabbit anti p-p38 -R Antibody (Tyr 182, sc-7975-R), p38 alpha Antibody (C-20, sc-535), p-ERK 1/2 -R Antibody (Thr 177, sc-16981-R), ERK 1/2 Antibody (H-72, sc-292838) -antibodies as well as goat anti-rabbit IgG-HRP (sc-2030) secondary antibody were from Santa Cruz Biotechnology, Inc (Santa Cruz, CA). 2,7-dichlorofluorescein diacetate (DCF-DA) was purchased from Sigma-Aldrich. All other reagents and chemicals were from (Sigma-Aldrich).

***Animals***

A total of 40 rats were obtained from Iranian Research Center (Pasteur Institute, Tehran) which included 10 young rats (4 month-old) and 30 rats in an age range of 13-15 months old. The rats were kept in the animal house for 10 days under natural conditions, in compliance with the cycle of daylight and darkness and the temperature of about 22 ^°^C in a box of polycarbonate which was provided them with free access to food and water. 

***Ethics statement***

Being all protocols in compliance with Helsinki Declaration was approved by the Ethics Committee of the University of Mohaghegh Ardabili. 

***Study design***

**Figure 1:**
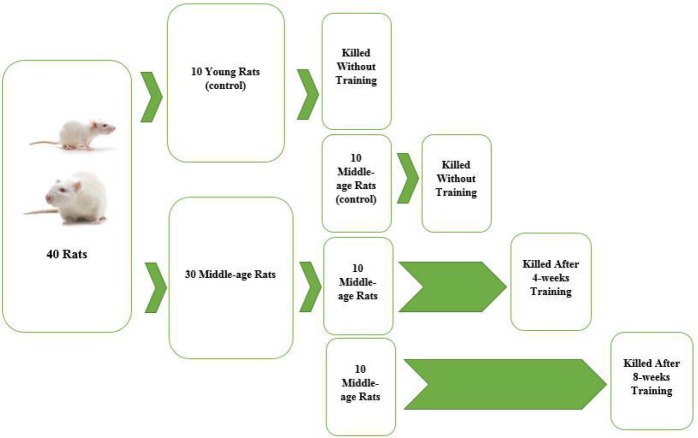
Forty rats including ten young rats and 30 middle-aged rats were prepared in this study. Ten young rats and 10 middle-aged rats (randomly) were labeled as a normal young control and middle-aged control respectively. The remaining 20 rats middle-aged have participated in an aerobic exercise program which randomly were trained for 4-week’s exercise (n=10) or eight weeks (n=10)

In the current experimental study, the statistical community consists of young rats (n=10, 4 months) and middle-aged male rats (n=30, 11-15 months). All young rats and 10 middle-aged rats (randomly) were labeled as a normal young control and middle-aged control respectively. The remaining 20 rats middle-aged have participated in an aerobic exercise program which randomly were trained for 4-week’s exercise (n=10) or 8 weeks (n=10) (Figure 1). The killings were carried out within 24 hr after the last training session. All rats were weighed in two times; upon entrance to study and exactly before killing. Throughout the sampling stage, blood samples were withdrawn from the heart and stored in -20 ^°^C to measure serum Klotho. The hearts were dissected and weighed before be subjected to other analyses. The heart tissue was stored either at -80°C for immunoblotting and fluorimetery or 10% formaldehyde to prepare the paraffin-embedded tissue sections.

***Exercise training protocol***

In the beginning, the rats were familiarized with the exercises on a rodent’s treadmill for 5 days (Technic Azma, Iran). Exercises began with an initial speed of 11 m/min, the slope of 0%, and distance traveled of 180 meters during the 13 min that reached to a speed of 14 m/min, the slope of 0%, and distance traveled of 460 meters during 34 min in the fourth week. Finally, in the eight weeks, they reached a speed of 16 m/min, the slope of 0%, and distance traveled of 830 meters during 54 min (33) (Table 1).

***Enzyme-linked immunosorbent assay (ELISA)***

The detection of Klotho protein in serum was performed using the corresponding ELISA kits (OKEH01598; Aviva Systems Biology Co, Ltd, CA, USA) based on standard sandwich ELISA technology. All steps of the protocol were done according to the manufacturer’s instruction. Briefly, the kit was equilibrated at room temperature before using. Seven standard wells were set on an enzyme-labeled coated plate. The standard curve was made in accordance with the optical density of serially diluted the lyophilized KL standard, subsequently, the samples were added to the wells. A biotinylated detector antibody specific for KL is added, incubated and followed by washing and adding an Avidin- Conjugated HRP. A chromogenic -enzymatic reaction was produced via catalyzing the TMB substrate which changed to yellow color after adding acidic stop solution. The optical density of yellow coloration was read at the wavelength of 450 nm by using a microplate reader (Stat Fax, Awareness Technologies, USA).

***Immunoblotting***

Western blotting for the expression of p38, p-p38, ERK1/2, and p- ERK1/2 in cardiac tissues was conducted using previously described methods (34) by a little modification. Briefly 100 mg frozen cardiac tissue was homogenized on ice using a rotor blender (Fisher) on ice-pre cold RIPA lysis buffer (50 mM Tris-HCl, pH 8.0, 0.1% sodium dodecyl sulfate, 150 mM sodium chloride, 0.5% sodium deoxycholate, and 1.0% NP-40) supplemented with protease and phosphatase inhibitor cocktail (Sigma Aldrich). Supernatants were obtained by centrifugation of crude homogenate at 12000 ×g for 20 min at 4 ^°^C by using froilabo SW14R (France). The protein concentration was detected using a Bradford assay kit (Sigma Aldrich). After mixing (1:1) with 2x sample loading buffer (Sigma Aldrich) and boiling for 5 min, 50 μg of protein were separated by electrophoresis on a denaturing SDS-PAGE. Proteins were then blotted onto PVDF Immobilin membrane. Ponceau red staining was used to confirm the transfer. The membranes were blocked for 1–2 hr in blocking buffer including 3% bovine serum albumin in phosphate-buffered saline and 0.1% Tween-20 (PBST). Membranes were subsequently probed with primary antibody (anti phospho-p38, p38α, p42/44 MAPK (REK1/2), phospho-p42/44 MAPK) diluted (1:500) in blocking buffer. After washing using PBST and then incubation with goat anti-rabbit secondary antibody for 1 hr at room temperature the antibody-antigen complex was detected using enhanced chemiluminescence (ECL; Amersham) with exposure to x-ray film (Fuji, Tokyo, Japan). Phospho-p38 and p-ERK1/2 were normalized against the total p38 and total ERK1/2 respectively.

***Measurement of intracellular H***_2_ ***O***_2_

Intracellular H_2_O_2 _in Cardiomyocytes were determined using a non-fluorescent dye DCF-DA (2′7-dichlorodihydrofluorescein diacetate) (Sigma-Aldrich, USA) which could be oxidized by ROS to the fluorescent dye DCF (2,7-dichlorofluorescin). Briefly, after deep anesthesia, animals were decapitated, hearts removed, and minced in ADS buffer (0.476% Hepes (w/v), pH 7.35, 0.1% glucose (w/v), 0.68% NaCl (w/v), 0.012% NaH_2_PO_4_ (w/v), 0.04% KCl (w/v), 0.01% MgSO_4_ (w/v)) including 0.4 mg/ml collagenase A (Roche) and 0.6 mg/ml pancreatin (Sigma-Aldrich) at 37 ^°^C with shaking. The cells were centrifuged at 1000 ×g and re-suspended in maintenance media (68% DMEM, 5% FCS, 10% horse serum, 17% Medium 199) and after adding 10μM DCF-DA incubated for 20 min at 37 ^°^C and 5% CO_2_. The fluorescence intensity of DCF was also measured with a fluorescence spectrometer (PerkinElmer, LS55, UK) at 24 ^°^C (excitation 488 nm, emission 525 nm, slit width 5 nm). The fluorescence intensity of DCF was normalized in the original cell suspension per mg protein (intensity of DCF /mgP).

***Histopathologic analyses and measuring of left heart dimensions***

The paraffin embedded and cut into 5-μm transverse sections heart tissue, were subjected to hematoxylin-eosin (H&E) and Masson trichrome staining. H& E slides were examined from point of view of morphologic changes and to measure the left ventricular wall thickness (LVWT) and left ventricular internal diameter (LVID). The dimensions of the heart were calculated using image analysis software (Image-Pro Plus v. 6.0; Image-Pro, USA). The Masson trichrome staining is performed to detect extracellular matrix formation such as collagen fibers which are index of fibrosis. Then were analyzed by Fiji software (http://imagej.nih.gov/; http://fiji.sc/Fiji)

***Statistical analysis***

Descriptive statistics were determined for all variables. The normal distribution of data was determined by the Kolmogorov-Smirnov test. Data were analyzed using appropriate Linear Mixed Model method (based on fixed effects) for the relationship between markers and Repeated-Measure design with Bonferroni *Post hoc* test for intragroup’ comparisons. Also, t-test analyses were used to compare differences between young control and middle-age control groups. Statistical significance was set at the alpha of α ≤0.05 for all tests. Analysis of all data was conducted using the Statistical Package for the Social Sciences 22 software (SPSS, Chicago, IL, USA).

## Results

***Klotho***

**Figure 2:**
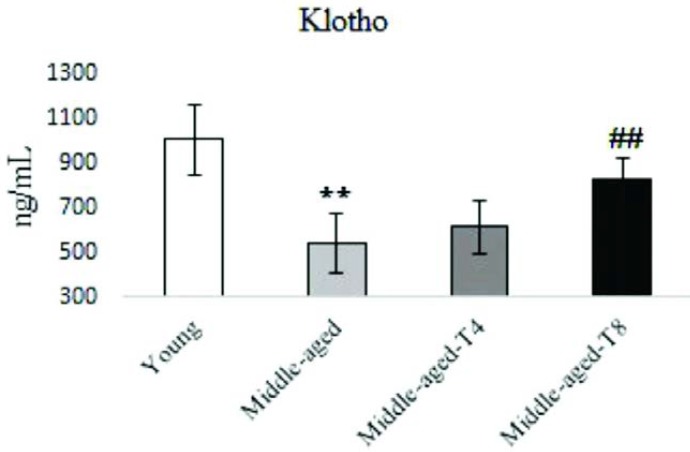
Serum levels of Klotho protein in young, middle-aged model, 4 weeks trained middle aged, and 8 weeks trained middle aged rats. Moderate exercise significantly increased Klotho in 8 weeks trained middle aged rats. **P<0.05 compared to young rats, ##*P<*0.05 compared to middle-aged rats

The middle-age condition decreased serum Klotho levels (*P*=0.001) versus control group, however moderate aerobic training increased serum levels of Klotho in middle-aged Wistar rats after eight weeks (*P*=0.011) (Figure 2). 

***ERK1/2 and P38***

**Figure 3:**
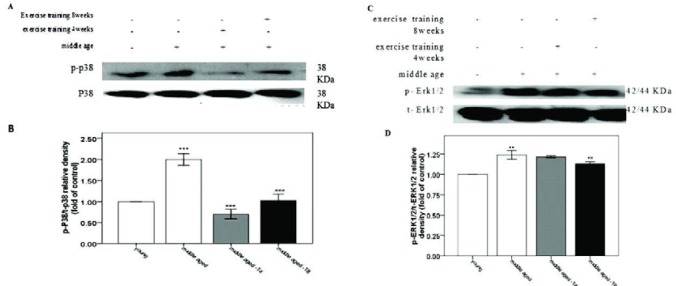
Phosphorylation of P38 MAPK protein in young, middle-aged model, 4 weeks trained middle aged, and 8 weeks trained middle aged rats. A, depicts the immunoblotting images of p38 and p-P38 MAPK. B. Bar chart shows the quantified protein bands of p-p38 normalized against p38 which is presented as fold of control. Moderate exercise reduced phospho P38 in both 4 weeks and 8 weeks trained middle-aged rats. C, depicts the immunoblotting images of ERK1/2 and p-ERK1/2 MAPK. B. Bar chart shows the quantified protein bands of and p-ERK1/2 normalized against ERK1/2 which is presented as fold of control. Moderate exercise reduced phospho ERK1/2 8 weeks trained middle-aged rats. ** *P<*0.05 compared to young rats, ## *P<*0.05 compared to middle-aged rats

**Figure 4:**
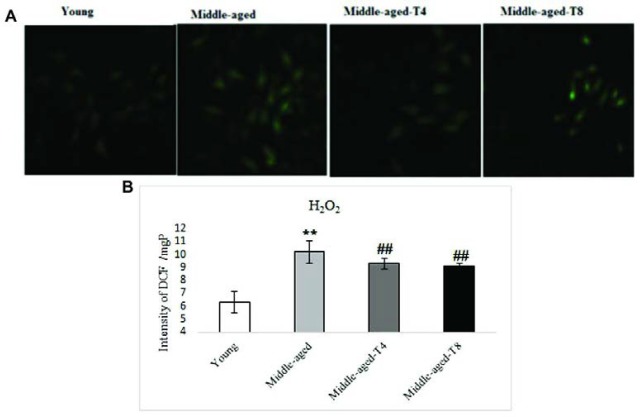
The levels of H_2_O_2_ in young, middle-aged model, 4 weeks trained middle aged, and 8 weeks trained middle aged rats. A, shows the amount of H_2_O_2_ qualitatively. By fluorescence specified. B, shows the quantified level of H_2_O_2_. Aerobic training significantly decreased H_2_O_2_ in the fourth and eighth weeks. ** *P≤ *0.05 compared to young rats, ## *P<* 0.05 compared to middle-aged rats

The middle-age condition increased phospho ERK1/2 (p-ERK1/2) (*P*=0.001), p-P38 (*P*=0.001) (Figure 3).and H_2_O_2 _(P=0.001) in heart tissue (Figure 4). But as shown in Figure 3 aerobic training significantly decreased p-ERK1/2 heart tissue levels after 8-weeks (*P*=0.001). However, moderate aerobic training decreased P38 in the fourth (*P*=0.01) and eight weeks (*P*=0.001) (Figure 3). 

***H***_2_ ***O***_2_

As a result of middle-age increased H_2_O_2 _(*P*=0.001) in heart tissue (Figure 4). However aerobic training significantly decreased H_2_O_2 _in the fourth (*P*=0.016) and eighth weeks (*P*=0.001) (Figure 4).

***Demographic morphologic changes in rat hearts***

**Figure 5:**
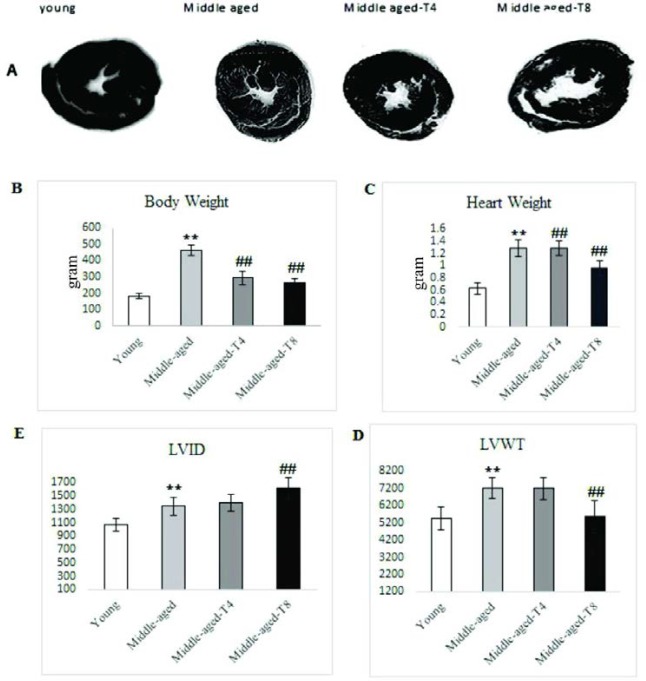
Effect of moderate exercise training on middle aged induced- morphologic changes in rat Hearts. A: Depicts scanned transverse sections. Note to the higher volume and lower walls thickness in trained rats in comparing the non-trained middle aged rats. B and C: Diagram changes of Heart Weight and Body weight. D. Left ventricular internal diameter (LVID). E: diagram changes of left Ventricular Wall Thickness (LVWT). ** *P<*0.05 compared to young rats, ## *P<* 0.05 compared to middle-aged rats

The middle-age condition increased heart weight (*P*=0.001) and body weight and led to pathological concentric hypertrophy, so that increased the LVWT (*P*=0.001) and LVID (*P*=0.001) (Figure 5). However, aerobic training significantly decreased body weight and heart weight after 4 and 8 weeks of aerobic exercise (*P*=0.001) (Figure 4). Also, Aerobic training increased LVID and decreased LVWT in middle-aged male Wistar rats after 8-week (Respectively, *P*=0.011 and *P*=0.028) (Figure 5).

***Fibrosis***

**Figure 6:**
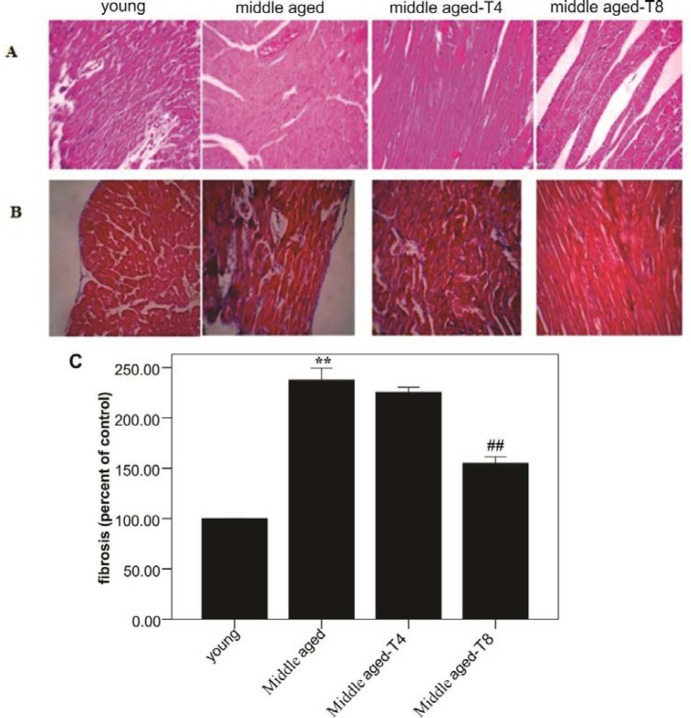
Effect of moderate exercise training on middle aged induced- cytopathology changes in rat hearts. A, Representative hematoxylin-and-eosin (HE)-stained thin slices of cardiac ventricles. Sections revealed a normal linear arrangement of myofibrils in young rat heart section. Note to massive and concentric fiber in aged rats heart tissues (40×). B, Representative Masson’s trichrome-stained sections from young, middle aged and 4 weeks and 8 weeks trained. Note and compare the accumulation of collagen fibers (blue) in the experimental group (40×). C, Shows the amount of fibrosis qualitatively

As shown in Figure 6, middle aged has increased fibrosis in rats, so that there was a significant difference in fibrosis between young and middle-aged rats (*P*=0.001). But exercise led to a significant decrease in fibrosis in the eighth week (*P*=0.001).

***Relationship between variables in middle-aged rats following aerobic training***

Also, we found that with increasing age from 4 months to 15 months in rats, there is a significant relationship between changes in serum Klotho and heart tissue levels of p-ERK1/2, p-P38 and H_2_O_2 _with LVID and LVWT. So that there was a significant relationship between serum Klotho with LVID (*P*=0.001) and LVWT (*P*=0.005). A similar relation was observed between H_2_O_2_ and fibrosis with LVID (*P*=0.001) and LVWT (*P*=0.001). Also, a significant relationship was observed between p-ERK1/2 with LVID (*P*=0.001) and LVWT (*P*=0.001), as well as between P38 with LVID (*P*=0.001) and LVWT (*P*=0.001) (Table 2).

We found that there was a significant relationship between serum Klotho with ERK1 / 2 (*P*=0.001), P38 (*P*=0.001), H_2_O_2 _(*P*=0.001), fibrosis (*P*=0.001) and the LVID (P=0.001) as a result of moderate aerobic training. Also, aerobic training had a significant correlation between H_2_O_2_ with ERK1/2 (*P*=0.001), P38 (*P*=0.001), fibrosis (*P*=0.001) and LVID (*P*=0.005). In addition, there was a significant relationship between ERK1/2 with LVID (*P*=0.001) and LVWT (*P*=0.002) as a result of moderate aerobic training. Also, exercise had a significant effect on the relationship between P38 with LVID (*P*=0.005), LVWT (*P*=0.018) and fibrosis (*P*=0.001). A similar significant relationship was observed between fibrosis with LVID (*P*=0.001) and LVWT (*P*=0.001) as a result of training (Table 3).

**Figure 7:**
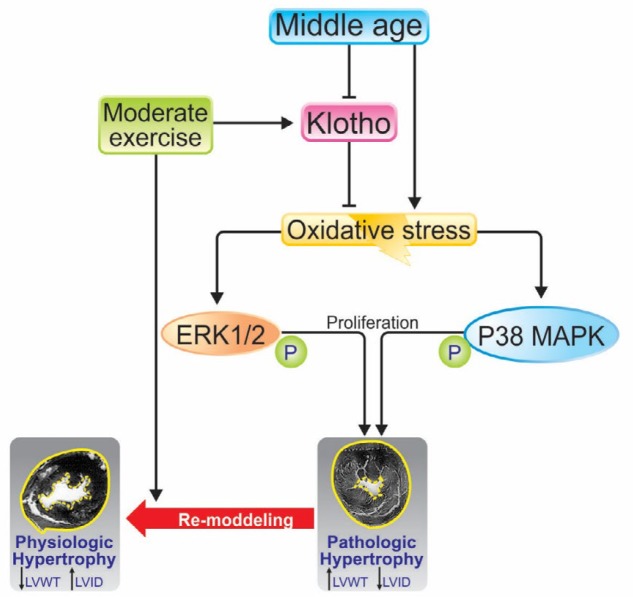
The mechanism of effect of exercise and middle-age on cardiac hypertrophy. Middle age is decreasing plasmatic Klotho and increases heart tissue oxidative stress. Increased oxidative stress is associated with increased amounts of p38 and ERK1.2, which leads to pathological hypertrophy. But exercise increases the amount of Klotho in middle-aged samples and provides a physiological hypertrophy

**Table 1:** Moderate-intensity aerobic exercise protocol

Week	Speed (m/min)	Slope (%)	Time (min)	Distance (m)
1	11	0	17	180
2	12	0	24	260
3	13	0	29	370
4	14	0	34	460
5	15	0	39	560
6	15	0	44	652
7	16	0	49	770
8	16	0	54	830

**Table 2:** The relationship between Klotho, H_2_O_2_, ERK1/2, and P38 with heart dimensions (from 4 to 15 months old).

Response	Variable	
	LVID	LVWT
	*P*-value #	*P*-value #
Klotho	0.001	0.005
H_2_O_2_	0.001	0.001
ERK1/2	0.001	0.001
P38	0.001	0.001
Fibrosis	0.001	0.001

# By liner mixed model (α≤0.05).

**Table 3:** The relationship between research variables with each other in middle-aged rats

Marker-1	Marker-2	*P*-value #
Klotho	H_2_O_2_	0.001**
	ERK1/2	0.001 **
	P38	0.001**
	LVID	0.001**
	LVWT	0.069
	Fibrosis	0.001 **
H_2_O_2_	P38	0.001**
	ERK1/2	0.001**
	LVID	0.005**
	LVWT	0.053
	Fibrosis	0.002 **
ERK1/2	LVID	0.001**
	LVWT	0.002**
	Fibrosis	0.139
P38	ERK1/2	0.001**
	LVID	0.005**
	LVWT	0.018**
	Fibrosis	0.001**
Fibrosis	LVID	0.001**
	LVWT	0.001**

# By liner mixed model (α≤0.05).

## Discussion

Increasing age leads to pathological hypertrophy (35). In the current study, the significance of modified heart weight and dimensions, the MAPK pathway signaling, fibrosis and ROS production in heart tissue in rats aged 15 months as middle-aged rats, were compared to 4-month-old rats as young rats. Then, the mentioned parameters were compared between the middle-aged group and moderate exercise-trained groups (4 and 8 weeks). We pointed that middle-age is associated with pathological hypertrophy and increased heart weight in male rats. On the other hand, significantly increased LVWT and LVID were due to middle-aged (LVWT increased far more than LVID) (Figure 7). 

Given the increase in the weight of the heart and LVWT, can be argued that concentric pathological hypertrophy occurs in middle-aged rats. Liao *et al.* (2015) also viewed heart weight gain of LVWT in older samples as concentric Hypertrophy (36). Consistent with this finding, Dedkov *et al.* (2014) also reported concentric development in middle-aged rats (37). The development of hypertrophy in older age groups of rats was accompanied by dramatic decreasing in plasmatic levels of Klotho protein. Kidney has been known as the main source of the Klotho protein (38). Reduction in plasma levels of Klotho in middle-aged rats can be attributed to kidney tissue damage or kidney failure. However, we did not assess renal status in the rats which may be considered as the limitation of the present study. It is likely that the changes in renal tissue were created by aging, leading to a decreased plasmatic levels of Klotho in rats (39-42). In terms of mechanisms, an increase in angiotensin-2 and oxidative stress is also a factor that reduces Klotho (30). In the current experiment, the increased intracellular H_2_O_2_ was observed in middle-aged heart tissues which may be extended to other tissues such as the kidney. Therefore, it may be assumed that the aging induced-oxidative stress is a major cause of renal damage and thus Klotho reductions. 

Reducing the amount of Klotho helps to induced autophagy (43), and increase in heart fibrosis (44). Recently scientists also revealed that oxidative stress and Klotho reduction could regulate p38 MAPK activity in mouse models of aging (45). In the current study, a significant increased p38 and ERK1/2 were observed in middle-aged rats heart tissues. Oxidative stress causes a wide range of hypertrophic kinase activity signals and transcription factors with MAPKs being one of them (46). That is why one of the important mechanisms for the involvement of oxidative stress in cardiac hypertrophy can be attributed to MAPKs. Other research has also demonstrated MAPKs activity and an increase in oxidative stress followed by mechanical stress in cardiomyocytes of neonatal rats (47). In another study, it was found that ventricular myocytes of adult rats affected by H_2_O_2_ increased the activity of P38 (48). For this reason, oxidative stress can probably be considered as a direct or indirect (by reducing Klotho) upstream regulator of MAPKs and a possible mechanism that could potentially impact the fibrosis and cardiac hypertrophy.

In addition, the correlation between increased LVID and LVWT with Klotho was shown in this experiment. Resultant data also showed that LVWT, fibrosis, intracellular H_2_O_2_, P-P38, and p-ERK1/2 were reduced as well as LVID and Klotho were increased after eight weeks of aerobic exercise. In the line with our results, previous studies explored that, by aerobic exercise, pathological hypertrophy in the sedentary middle-aged rats had changed to eccentric physiological hypertrophy (29). However, their research was not based on middle-aged rats and they did not express a clear mechanism for their findings. Our observations in this study forced us to assume that the aerobic exercise training-induced changes in the heart tissue may partially result from downregulation of MAPKs which were affected by the decreased ROS or elevated serum levels of Klotho protein regardless of its source.

As already stated, significantly increased the intracellular levels of H_2_O_2_ were found in the heart tissues of middle-aged rats compared to young rats, but moderate-intensity aerobic exercise lead to the significant reduction in H_2_O_2_ and the increase in plasmatic Klotho levels which may be explained as follows. Exercise training could decrease the accumulation of ROS and probable renal injury and thus restored Klotho levels which in turn reduces intracellular H_2_O_2_ in heart tissue. It is possible that exercise can slow the aging process (49), and Klotho’s role in the reduction of H_2_O_2_ can be explained by the increased activity of catalase and the regulation of SOD-2 gene expression (50, 51). In the previous study, we have shown that aerobic exercise leads to the increased expression of superoxide dismutase 2 (SOD2) and decreased malondialdehyde (MDA) in middle-aged men (52). Accordingly, reduction of H_2_O_2_ by aerobic exercise can be attributed to an increase Klotho and antioxidant enzymes. With regard to the mechanism of how exercise affects an increase of Klotho, the roles of peroxisome proliferator-activated receptor-γ (PPARγ) and angiotensin-2 should be noted (30, 53, 54).

In addition, the correlation between LVWT and LVID with H_2_O_2_ was shown in this experiment. Also, there was a significant association between p-P38 and p-ERK1/2 with LVWT and LVID as. result of moderate training. There are several mechanisms for the impact of oxidative stress on cardiac hypertrophy (55, 56). According to other studies, extracellular stimulating factors cause increases of cardiac hypertrophy through the phosphorylation of ERK1/2 signals (57, 58). Stimulating both receptors can cause hypertrophic signaling pathways through Ras-ERK (59). ERK1/2 becomes active also through the setting of positive and negative feedback from other components of the MAPKs signaling network. This process includes a negative adjustment of ERK by other MAPKs (60). Our findings also showed a significant correlation between P38 and p-ERK1/2 as result of exercise. Therefore, one of the roles of oxidative stress on ERK1/2 can be considered from this point of view. In other words, the decrease in the oxidative stress caused by exercise leads to a decrease in ERK1/2 levels by reducing P38. As in our study, the reduction of H_2_O_2_, ERK1/2 and P38 as result of moderate training was also associated with decreased LVWT and fibrosis and increased LVID in trained middle-aged rats.

Overall, the mechanism between the variables in this study can be described in the following manner. Oxidative stress caused by middle-age and a sedentary lifestyle is able to activate MAPKs through apoptosis signal-regulating kinase-1 (ASK) and can increase the activities of MAPKs. ASK1 or kinase-5 kinase MAPKs (MAP3K5) is able to activate MAPKs as well as P38 in response to factors such as oxidative stress. Moreover, it has been found that Klotho should be considered, including factors contributing to deactivation of ASK1 (45). Therefore, it is likely that exercise (and consequently increased Klotho), through the increase of antioxidants and reduced H_2_O_2_, leads to a reduction in ASK1 and consequently P38 (31). On the other hand, H_2_O_2_ is able to activate ERK1/2 through the Raf-Ras pathway, as well as tyrosine kinase pathways including PDFG-beta receptor and Src and protein kinase C- delta (PKC-delta) (61). 

It seems that exercise and consequent Klotho can be effective in reducing ERK1/2 through an increase in antioxidants, reduced H_2_O_2_ and subsequent inhibition of the Raf-Ras pathway. Although it is also likely that Klotho effects PDGF-beta, PKC-delta and Src. However, research in this area is not currently available. Alternatively, Klotho can reduce TNF-a (factor increase of P38) and thereby also contribute to reducing the amount of P38 (62, 63). 

Despite this, it is a possible and important effect of P38 on ERK1/2 as well. P38 is able to increase the activity of ERK1/2 (60) so there is a reduction in the amount of P38 in trained middle-aged rats by decreased oxidative stress, and increased Klotho is effective in the reduction of ERK1/2. Our findings showed a significant relationship between them (P38 and ERK1/2), in line with the decrease in P38, was reduced the amount of ERK1/2 (Figure 7). 

## Conclusion

The resultant data indicate that moderate-intensity aerobic exercise can be effective in converting pathological hypertrophy to physiological hypertrophy in middle-aged rats. It seems aerobic exercise effect may be partially mediated by increasing Klotho and reducing oxidative stress as well as reducing ERK1/2, P38 and fibrosis.
